# Acromegaly and Metabolic Dysfunction–Associated Steatotic Liver Disease: Clinical and Therapeutic Implications

**DOI:** 10.1007/s13679-026-00747-y

**Published:** 2026-08-05

**Authors:** Stergios A. Polyzos, Alessandro Mantovani, Giovanni Targher

**Affiliations:** 1 Laboratory of Pharmacology, School of Medicine, Aristotle University of Thessaloniki, Thessaloniki, Greece; 2 Department of Medicine, University of Verona, Verona, Italy; 3 Metabolic Diseases Research Unit, IRCCS Sacro Cuore – Don Calabria Hospital, Negrar di Valpolicella, Italy

**Keywords:** Acromegaly, Growth hormone, Insulin–like growth factor, Metabolic dysfunction–associated steatohepatitis, Metabolic dysfunction–associated steatotic liver disease, Pegvisomant

## Abstract

**Purpose of Review:**

This narrative review aims to critically summarize available data on the association between adult acromegaly and metabolic dysfunction–associated steatotic liver disease (MASLD), with a particular focus on clinical associations and treatment implications.

**Recent Findings:**

From a pathophysiological perspective, growth hormone (GH) may improve hepatic steatosis, inflammation, and fibrosis by acting directly on hepatocytes and indirectly (through insulin–like growth factor–1) on hepatic stellate cells, thereby inducing their senescence. Nonetheless, GH excess may also favor the development of hepatocellular carcinoma, possibly through mechanisms unrelated to hepatic fibrosis. From a clinical perspective, individuals with acromegaly have low rates of hepatic steatosis and visceral adipose tissue (VAT), but high insulin resistance (IR), which contradicts the generally observed positive association between IR and VAT or hepatic steatosis. By contrast, limited data do not support lower rates of hepatic fibrosis in acromegaly, possibly because GH excess is controlled after diagnosis. From a therapeutic perspective, published evidence, derived mainly from case series, suggests that surgical or pharmacological management of acromegaly increases hepatic steatosis and VAT, despite decreasing IR. However, the long–term effect of acromegaly control on hepatic inflammation and fibrosis remains largely unknown.

**Summary:**

Acromegaly is associated with IR, type 2 diabetes mellitus, hypertension and cardiovascular disease; however, acromegaly is inversely associated with VAT and MASLD. Further mechanistic and clinical studies are warranted to better elucidate the intriguing association between acromegaly and MASLD.

## Introduction

Previously known as nonalcoholic fatty liver disease (NAFLD), metabolic dysfunction–associated steatotic liver disease (MASLD) affects more than one–third of adults worldwide, and pharmacological options remain limited [1, 2]. MASLD encompasses a phenotypic continuum, ranging from isolated hepatic steatosis, which may progress to metabolic dysfunction–associated steatohepatitis (MASH), previously termed nonalcoholic steatohepatitis (NASH), and subsequently to fibrosis, cirrhosis, and hepatocellular carcinoma (HCC) in a small percentage of cases. However, given the high prevalence of this metabolic liver disease, these outcomes affect a large absolute number of individuals [3]. The pathophysiology of MASLD is multifactorial and varies among affected individuals [4], highlighting the need for a patient–specific management strategy [5]. In this setting, multiple, apparently diverse factors may contribute to the pathophysiology of MASLD, including dietary habits, physical inactivity, hormones, adipokines, hepatokines, cytokines, gut microbiota, infections, and endocrine disruptors [6]. In parallel, MASLD is a multisystem disease, closely associated with extrahepatic morbidity, including obesity, type 2 diabetes mellitus (T2DM), atherogenic dyslipidemia, arterial hypertension, cardiovascular diseases (CVD), chronic kidney disease, and extrahepatic cancers [7]. Apart from obesity and T2DM, other metabolic and endocrine diseases have also been associated with MASLD, such as primary hypothyroidism, hypogonadism, polycystic ovary syndrome, Cushing’s syndrome, and growth hormone (GH) deficiency (GHD) [8, 9].

We previously summarized evidence supporting an association between adult GHD and MASLD [10, 11]. Indeed, MASLD is highly prevalent in adults with GHD [11]. It was recently shown that low insulin–like growth factor–1 (IGF–1), a downstream product of GH receptor signaling in hepatocytes, was associated with liver cirrhosis and proposed as a predictor of liver–related mortality [12]. Acromegaly lies at the opposite end of the spectrum from GHD, as it is characterized by GH excess. It is a rare endocrine disease, usually caused by pituitary adenomas arising from adult somatotroph cells (somatotroph adenomas) [13]. In adults, acromegaly is typically characterized by progressive dysmorphic facies, macroglossia, mandibular overgrowth, dental gaps, prognathism, and progressive enlargement of the hands and feet [13]. Because GH excess affects multiple organs, some affected individuals develop IR, T2DM, arterial hypertension, and CVD [13], conditions that, as mentioned above, are strongly associated with MASLD [7, 14]. Nevertheless, most observational studies report low rates of MASLD in adults with acromegaly [10], making the association between acromegaly and MASLD particularly intriguing.

Based on these considerations, this narrative review aims to critically summarize available data on the association between adult acromegaly and MASLD, with a particular focus on clinical associations and treatment implications.

## Literature Search

A literature search was conducted in PubMed with no publication date restrictions. Our electronic search strategy combined Medical Subject Heading (MeSH) terms and non–MeSH terms, yielding the following search string, which formed the core of our electronic search: ((Acromegaly[Mesh]) OR (Growth Hormone–Secreting Pituitary Adenoma[Mesh]) OR (Growth Hormone[Mesh]) OR (Growth Hormone–Releasing Hormone[Mesh]) OR (Insulin–Like Growth Factor I[Mesh]) OR (Insulin–Like Growth Factor II[Mesh]) OR (Insulin–Like Growth Factor Binding Proteins[Mesh]) OR (“somatotroph adenoma”) OR (“inappropriate growth hormone secretion syndrome”) OR (“somatotropin hypersecretion”) OR (“somatostatin receptor analog*”) OR octreotide OR lanreotide OR pasireotide OR (“GH receptor antagonist”) OR (“growth hormone receptor antagonist”) OR pegvisomant OR cabergoline) AND ((Non–alcoholic Fatty Liver Disease[Mesh]) OR (“nonalcoholic fatty liver disease”) OR (“nonalcoholic steatohepatitis”) OR (“non–alcoholic fatty liver disease”) OR (“nonalcoholic steatohepatitis”) OR (“non–alcoholic steatohepatitis”) OR NAFL OR NASH OR NAFLD OR MAFLD OR (“metabolic dysfunction associated fatty liver disease”) OR (“metabolic associated fatty liver disease”) OR (“metabolic dysfunction associated steatotic liver disease”) OR MASLD OR MASH OR (“steatotic liver disease”) OR SLD). Using this search string, 245 articles were initially retrieved (on February 23, 2026). The same search strategy was repeated on June 1, 2026 (before the initial submission), yielding 250 articles. Of them, 39 were selected for providing the best available evidence. Furthermore, 9 additional articles were identified from the references of the selected articles. Because this is a narrative review, articles beyond the predefined search results were included at the authors’ discretion, if deemed pertinent to the topic.

## Pathophysiology

**Fig. 1:**
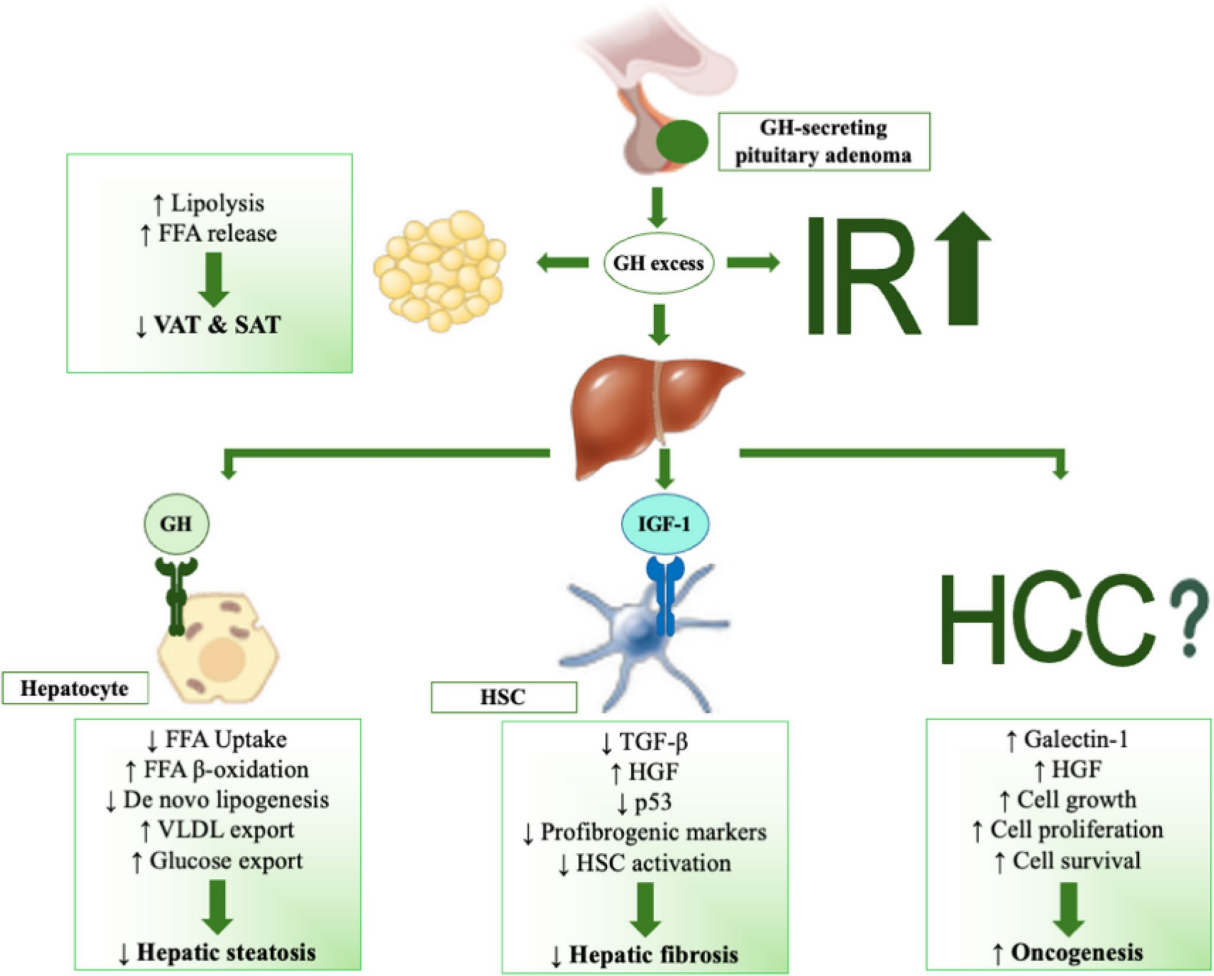
The intriguing effects of GH on the liver, adipose tissue, insulin resistance, and hepatocarcinogenesis. In acromegaly, GH excess appears to reduce hepatic steatosis in hepatocytes by decreasing FFA uptake, increasing FFA β–oxidation, reducing hepatic *de novo* lipogenesis, and increasing VLDL and glucose export. In HSCs, high IGF–1 appears to prevent hepatic fibrosis by decreasing TGF–β, increasing HGF, and suppressing p53, thereby reducing HSC activation. Despite these seemingly favorable hepatic effects, GH excess may also promote HCC development by upregulating galectin–1 and HGF, thereby increasing cell growth, proliferation, and survival. VAT and SAT are also reduced in acromegaly, mainly due to GH–induced lipolysis. Paradoxically, IR increases in acromegaly. These changes are reversed after surgical and/or pharmacological management of acromegaly. Abbreviations: FFA, free fatty acids; GH, growth hormone; HCC, hepatocellular carcinoma; HGF, hepatic growth factor; HSCs, hepatic stellate cells; IGF–1, insulin–like growth factor–1; IR, insulin resistance; SAT, subcutaneous adipose tissue; TGF–β, transforming growth factor–β; VAT, visceral adipose tissue; VLDL, very low–density lipoprotein

GH is released in a pulsatile manner by somatotroph cells of the anterior pituitary; it stimulates IGF–1 production in the liver, which primarily mediates its systemic effects [10]. GH is mainly regulated by GH–releasing hormone (GHRH) and somatostatin, which stimulate and inhibit GH secretion, respectively [10]. The metabolic effects of GH largely antagonize insulin actions, thereby inducing systemic and hepatic IR [10]. Consequently, adults with acromegaly have IR but, unexpectedly, reduced visceral adipose tissue (VAT) mass and intrahepatic lipid (IHL) [15]. This contradicts the positive associations between IR and VAT or IHL observed in the general population and in specific patient populations, including individuals with MASLD [16], T2DM [17], or lipodystrophy [18]. In contrast to decreased VAT and IHL, T2DM, arterial hypertension and dyslipidemia (characterized by high triglycerides and low high–density lipoprotein–cholesterol concentrations) are common manifestations of both MASLD [17] and acromegaly [19, 20] (Fig. 1).

### Effects of GH on Hepatic Steatosis

In hepatocytes, GH acts directly by binding the growth hormone receptor (GHR) on the plasma membrane, primarily activating the Janus kinase (JAK)2–signal transducer and activator of transcription (STAT)5 signaling pathway, as reviewed in detail elsewhere [10]. Briefly, GH leads to: a) stimulation of glycogenolysis and gluconeogenesis, thereby increasing hepatic glucose production; b) reduced free fatty acid (FFA) uptake by hepatocytes; c) reduced hepatic de novo lipogenesis; d) more efficient fatty acid (FA) β–oxidation in hepatic mitochondria; and e) enhanced export of very low–density lipoprotein (VLDL) from hepatocytes, thereby increasing hepatic triglyceride clearance [21–24]. These effects reduce IHL, improving hepatic steatosis [10] (Fig. 1). Importantly, the effects of GH on hepatocytes are not mediated through IGF–1 [10, 25].

### Effects of GH on Hepatic Inflammation and Fibrosis

In contrast to the direct effect of GH on hepatocytes, its effect on hepatic stellate cells (HSCs), the key cells driving hepatic fibrosis, appears to be mediated by IGF–1 [26]. Indeed, HSCs express IGF–1 receptors, through which IGF–1 induces HSC senescence and, therefore, reduces hepatic fibrosis in MASH mouse models [26]. Furthermore, IGF–1 ameliorates mitochondrial function and decreases oxidative stress, thereby mitigating hepatic inflammation and fibrosis [26]. These effects are not observed in p53 knock–out mice, which are considered to play a crucial role in HSC senescence [26], thereby suggesting that IGF–1 may, at least in part, exert its effects on HSCs by suppressing p53. Other authors also showed that IGF–1 gene transfer to the rat liver attenuates carbon tetrachloride–induced hepatic steatosis and fibrosis and reduces oxidative stress–induced hepatocyte senescence by inhibiting the nuclear interaction between p53 and progerin [27]. Decreased activation of HSCs induced by carbon tetrachloride, thereby reducing hepatic fibrosis, is also observed in transgenic mice overexpressing IGF–1. In this study, the effects of IGF–1 are partly driven by suppression of transforming growth factor–β (TGF–β) and induction of hepatocyte growth factor (HGF) [28]. Other authors also reported that mice treated with mesenchymal cells (MSCs) overexpressing IGF–1 exhibit attenuation of hepatic fibrosis induced by thioacetamide or bile duct ligation, along with upregulation of hepatic IGF–1 and HGF [29] (Fig. 1).

A critical question is whether GH and/or IGF–1 may affect Kupffer cells, which play a key role in hepatic inflammation. Experimental evidence indicates that GH [30, 31] and IGF1 [32] can promote a phenotypic switch in macrophages from M1 to M2, thereby potentially conferring an anti–inflammatory effect, although this has not been shown specifically in Kupffer cells. Other authors also reported that mice treated with MSCs overexpressing IGF–1 exhibit upregulation of IGF–1 and HGF in hepatic macrophages, reduced oxidative stress, and decreased profibrogenic and HSC activation markers [33]. These changes suggest that IGF–1 may lead to a shift toward an anti–inflammatory phenotype in hepatic macrophages and a less–activated phenotype in HSCs.

### Effects of GH on Hepatocarcinogenesis

Despite the anti–steatotic, anti–inflammatory, and anti–fibrotic effects of GH, persistently elevated GH may predispose to HCC in mouse models. Specifically, transgenic mice overexpressing GH exhibit hepatocyte hypertrophy, with enlarged nuclei and accelerated proliferation; these effects are mediated by pathways involved in cell growth, proliferation, and survival [34]. Deletion of STAT5 appears to predispose to HCC development in mice exposed to carbon tetrachloride, even without severe hepatic fibrosis. This is attributed to upregulation of TGF–β and STAT3 signaling in hepatocytes induced by loss of STAT5 function [35]. Other authors also reported that transgenic mice combining GH overexpression and STAT5 deletion develop HCC earlier and more advanced than mice overexpressing GH without STAT5 deletion, despite reduced chronic inflammation and lower premature mortality in the former than in the latter [36]. Given that GH signaling in hepatocytes is largely mediated by the JAK2–STAT5 pathway, these findings appear counterintuitive and highlight the need for further mechanistic studies to determine whether hepatic inflammation and fibrosis can become dissociated from HCC development in the context of sustained GH excess. 

The links between GH excess and HCC remain incompletely elucidated. Galectin–1 may be one such link, as it was upregulated early in the life of transgenic mice overexpressing GH, remained persistently upregulated during aging, and was markedly upregulated when HCC developed [37]. Similarly, human HCC cells treated with GH showed increased galectin–1 expression [37]. Yet further mechanistic studies are needed to clarify the intriguing connections among GH, galectin–1, and hepatocarcinogenesis. In line with the pathophysiologic role of the GH/IGF–1 axis in the development and progression of HCC, pharmacological interventions targeting the IGF–1 receptor for HCC management are under investigation in clinical trials. These include monoclonal antibodies against the IGF–1 receptor (e.g., ganitumab, figitumumab, dalotuzumab), tyrosine kinase inhibitors targeting the IGF–1 receptor (e.g., linsitinib), and antibodies against HGF (e.g., ficlatuzumab), as reviewed elsewhere in detail [38].

Collectively, GH may reduce hepatic inflammation and fibrosis both indirectly, by attenuating hepatic steatosis, thereby limiting progression to advanced disease, and directly, by inducing HSC senescence, an effect primarily mediated by IGF–1. Nonetheless, beyond these seemingly favorable hepatic effects, GH excess may also promote HCC development through mechanisms that may be unrelated to hepatic fibrosis (Fig. 1). These findings may have clinical and therapeutic implications and warrant further research. 

## Association of Acromegaly with MASLD: Evidence from Observational Studies

Relevant observational studies assessing the association between acromegaly and MASLD are summarized in Table 1 [39–49]. All retrieved studies were cross–sectional or case–control, and no relevant cohort studies were identified. Study populations were from Europe, North America, and Asia (Table 1). The first relevant study was published in 2008 [39]. In all retrieved observational studies, MASLD diagnosis was based on imaging methods and non–invasive tests (NITs) for hepatic steatosis and fibrosis. Importantly, advanced imaging techniques, such as magnetic resonance imaging–proton density fat fraction (MRI–PDFF), magnetic resonance spectroscopy (MRS), and magnetic resonance elastography (MRE), were used in many of these studies (Table 1). In contrast, liver biopsy was not performed in any study. It should be underlined that magnetic resonance (MR) techniques, including MRI–PDFF and MRS (used for the assessment of hepatic steatosis) and MRE (used for the assessment of hepatic fibrosis), offer higher accuracy and reproducibility compared with other imaging (e.g., ultrasound-based techniques or computed tomography) or non-imaging NITs [50]. However, the higher cost and lower availability of MR techniques currently limit their widespread use in clinical practice [50].

**Table 1:** MASLD in patients with acromegaly: evidence from observational studies*

First author, Year, Origin [Reference]^†^	Study design	Study groups (*n*)	BMI (kg/m^2^)^§^	Method(s) of diagnosis of NAFLD/MAFLD/MASLD	Metrics for HS and/or fibrosis	Main findings
Szendroedi 2008 Spain [39]	Case–control	Patients with controlled acromegaly (7) vs. healthy controls (7)	27.8 ± 1.3 vs. 25.6 ± 1.5^∫^	MRS (IHL)	IHL (%): about 2.5 vs. 7.5^a^	Lower IHL (about 3–fold) in patients with controlled acromegaly than controls.
Winhofer 2014 Austria [40]	Case–control	Patients with active acromegaly (10) vs. healthy controls (12)	27.9 ± 5.3 vs. 27.0 ± 3.4	MRS (IHL)	IHL (%): 1.2 ± 1.2 vs. 4.3 ± 3.5^a^	Lower IHL (about 3–fold) in patients with active acromegaly than controls.
Bredella 2017 USA [41]	Case–control	Patients with active acromegaly (20) vs. healthy controls (20)	30.4 ± 5.4 vs. 30.5 ± 5.9	MRS (IHL)	IHL (%): 2.0 (1.0–4.0) vs. 6.0 (2.0–22.0)^a^	Lower IHL (about 3–fold) in patients with active acromegaly than controls. Higher glucose, insulin and HOMA–IR in patients than controls.
Koutsou-Tassopoulou 2019 Germany [42]	Cross–sectional	Patients with acromegaly (20): presence of *PNPLA3* p.I148M risk allele (M) (8) vs. absence of *PNPLA3* p.I148M risk allele (M) (12)	31.3 ± 4.8 vs. 30.1 ± 3.9	TE (CAP: HS) TE (LS)	HS (%): 50.0 (total) LS (F ≥ 1; %): 27.3 (total)	Lower CAP in the absence of *PNPLA3* p.I148M risk allele (M). Similar LS in the presence or absence of *PNPLA3* p.I148M risk allele (M).
Fellinger 2020 Austria [43]	Case–control	Patients with active acromegaly (15) vs. healthy controls (17)	26.6 ± 5.1 vs. 26.8 ± 5.0	MRS (IHL)	IHL (%): about 2.0 vs. 3.0^a^	Lower IHL in patients with active acromegaly than controls. Higher ATP and lower carnitine species in patients than controls. Lower UI in patients than controls. Similar LFTs in patients and controls. Higher HOMA–IR in patients than controls.
Kuker 2021 USA [44]	Case–control	Patients with active acromegaly (8 of 21 subjected to hepatic MRS) vs. healthy controls (7)	29.9 (23.2–40.8) vs. NA^ƒ^	MRS (IHL)	1.8 (0.7–5) vs. 3.0 (1.6–13.2)^ƒ,a^	Lower IHL in patients with active acromegaly than controls.
Arlien-Søborg 2023 Denmark [45]	Case–control	Patients with untreated (active) acromegaly (21) vs. patients with GHD (12) vs. healthy controls (12)	27.9 (26.0–29.7) vs. 26.7 (24.2–29.1) vs. 27.1 (24.6–29.5)^∂^	MRS (IHL)	1.8 (1.0–3.2) vs. NA vs. NA^a^	Lower IHL in patients with active acromegaly than controls.
Eroglu 2023 Turkey [46]	Case–control	Patients with active acromegaly (15) vs. controlled acromegaly (17) vs. controls (19)	29.6 (27.0–30.9) vs. 31.6 (27.6–32.1) vs. 29.4 (27.7–31.6)^#^	MRI–PDFF (HS) MRE (LS) FLI, HSI, TyG (HS) FIB–4, APRI, NFS (fibrosis)	HS (%): 0.0 vs. 58.8 vs. 26.3^a^ LS (%): 21.4 vs. 23.5 vs. 10.5	Lower rates of HS in patients with active than controlled acromegaly. Similar rates of LS in patients and controls. Lower FLI and TyG in patients with active than controlled acromegaly, but not than healthy controls. Higher FLI and TyG in patients with controlled acromegaly than healthy controls. Similar HSI, FIB–4, APRI, NFS between groups. TyG was positively correlated with HS (MRI-PDFF) in all groups. FLI and HSI were positively correlated with HS (MRI-PDFF) in the control group, but were not in patients with active or controlled acromegaly.
Coskun 2024 Turkey [47]	Case–control	Patients with active acromegaly (23) vs. healthy controls (46)	27.3 (26.7–30.0) vs. 28.0 (25.8–30.6)^#^	QUS (TAI: HS) SWE (LS)	HS (%): 21.6 vs. 52.2^a^ LS (F ≥ 2; %): 13.3 vs. 0.0^a^	Lower rates of HS, but higher rates of presumed fibrosis (F ≥ 2), in patients with active acromegaly than controls. Higher glucose, insulin and HOMA–IR in patients than controls. Similar ALT, AST, GGT in patients and controls.
Yamamoto 2025 Japan [48]	Cross–sectional	Patients with untreated (active) acromegaly (66): BMI < 25 kg/m^2^ (39) vs. BMI ≥ 25 kg/m^2^ (27)	22.7 (20.0–24.1) vs. 27.6 (25.9–29.8)^#^	US (HS)	HS (%): 8.8 vs. 29.1 17.2 (total)	Lower rates of HS in patients with < 25 than ≥ 25 kg/m^2^.
Apaydin 2026 Turkey [49]	Case–control	Patients with acromegaly (58) vs. controls with metabolic diseases (61) vs. healthy controls (82)	30.4 ± 5.4 vs. 32.2 ± 6.5 vs. 26.6 ± 5.1^a^	TE (CAP: HS) TE (LS)	HS (%): 36.2 vs. 68.8 vs. 27.7^a^ LS (F ≥ 2; %): 5.1 vs. 14.7 vs. 7.3	Lower CAP in patients with acromegaly than controls with metabolic diseases, but not than healthy controls. Lower LS in patients with acromegaly than controls with metabolic diseases, but similar rates of presumed fibrosis (F ≥ 2) between groups. Not significantly different CAP or LS between patients with active (*n* = 13) and controlled (*n* = 45) acromegaly.

*ALT* alanine aminotransferase, *APRI* aspartate aminotransferase to platelet ratio index, *AST* aspartate aminotransferase, *ATP* adenosine triphosphate, *BMI* body mass index, *CI* confidence interval, *CAP* controlled attenuation parameter, *F* fibrosis stage, *FIB–4* fibrosis–4 index, *FLI* fatty liver index, *GGT* gamma glutamyltransferase, *GHD* growth hormone deficiency, *HOMA–IR* homeostasis model assessment–insulin resistance, *HS* hepatic steatosis, *HSI* hepatic steatosis index, *IHL* intrahepatic lipid, *LFTs* liver function tests, *LS* liver stiffness, *MAFLD* metabolic dysfunction–associated fatty liver disease, *MASLD* metabolic dysfunction–associated steatotic liver disease, *MRE* magnetic resonance elastography, *MRI–PDFF* magnetic resonance imaging–proton density fat fraction, *MRS* magnetic resonance spectroscopy, *n* number, *NA* not available, *NAFLD* nonalcoholic fatty liver disease, *NFS* NAFLD fibrosis score, *PNPLA3* patatin–like phospholipase domain–containing protein 3, *QUS* quantitative ultrasonography, *SD* standard deviation, *SWE* shear wave elastography, *TAI* tissue attenuation imaging, *TE* transient elastography, *TyG* triglyceride to glucose index, *UI* unsaturation index, *US* ultrasonography

*: Original nomenclature of the liver disease (i.e., NAFLD, MAFLD, MASLD) was retained in the table for each study, for the sake of consistency

†: Studies are sorted in alphabetical order, primarily according to the publication year and secondarily according to the first author surname

a: Statistically significant between groups

§: Data presented as mean ± SD, except if it is differently indicated

∫: Mean ± standard error of the mean

∂: Median (95% CI)

ƒ: Median (range)

#: Median (25th 75th percentile)

### Association Between Acromegaly and Hepatic Steatosis

In most studies, IHL was lower in patients with acromegaly than in those without acromegaly (Table 1). Rates were approximately 1.5– to 2–fold lower in acromegaly and up to 3–fold lower in some studies using MRS [39-41] (Table 1). Evidence comparing patients with active (uncontrolled) and controlled acromegaly is controversial: one study reported lower rates of hepatic steatosis in active than in controlled acromegaly [46], but another did not [49]; it should be noted that different imaging methods were used to assess hepatic steatosis in these studies (MRI-PDFF and transient elastography [TE], respectively). Notably, lower rates of hepatic steatosis or lower IHL were observed despite higher IR among patients with acromegaly [41, 43, 47]. Importantly, IR is a key pathogenic factor in MASLD, including the development of hepatic steatosis and progression to advanced phenotypes (MASH, hepatic fibrosis) [16]. 

Lower rates of hepatic steatosis were also observed in patients with acromegaly who lacked the p.I148M risk allele (M) of the gene *patatin–like phospholipase domain–containing protein 3* (*PNPLA3*) [42], a single–nucleotide polymorphism closely correlated with the presence and severity of MASLD [51]. Body mass index (BMI), a major confounding factor in MASLD studies, was similar across groups in all but one study [49] (Table 1). As expected, hepatic steatosis rates were higher in patients with acromegaly and higher BMI [48], as well as in those with coexisting T2DM [49]. 

Despite lower rates of hepatic steatosis or lower IHL in acromegaly, liver function tests (LFTs) did not differ significantly between patients with acromegaly and controls [43, 47], suggesting that LFTs do not reliably reflect the presence and severity of hepatic steatosis in patients with acromegaly, as they may do in patients with hepatic steatosis or MASH in the general population [52]. Similarly, NITs for hepatic steatosis were poorly correlated with hepatic steatosis (as assessed by MRI–PDFF) in patients with acromegaly [46]. In a study using lipidomics and metabolomics, lower carnitine species were observed in patients with active acromegaly compared with controls, indicating more efficient mitochondrial β–oxidation in patients with acromegaly [43]. This may partly explain the lower rates of hepatic steatosis or lower IHL observed in acromegaly. A limitation of all studies is their small sample size, largely owing to the rarity of acromegaly.

### Association Between Acromegaly and Hepatic Fibrosis

Evidence on hepatic fibrosis in patients with acromegaly is very limited. Liver stiffness (LS; measured by MRE) was similar among patients with active or controlled acromegaly and controls [46], suggesting that hepatic fibrosis was unaffected by disease status or control. In the same study, NITs for hepatic fibrosis were also similar across groups [46]. In another study [49], similar rates of presumed moderate hepatic fibrosis (assessed by TE) were observed in patients with acromegaly and healthy controls, as well as between patients with active and controlled acromegaly. Conversely, a study that measured LS using shear–wave elastography reported higher rates of presumed moderate hepatic fibrosis among patients with active acromegaly than among controls [47].

### Critical Summary on Observational Data Linking Acromegaly with MASLD

IHL or the rates of hepatic steatosis are lower in patients with acromegaly than in controls without acromegaly, despite higher IR in the former group. Other risk factors for hepatic steatosis, including higher BMI and the presence of the *PNPLA3* p.I148M risk allele, appear similar to those observed in unselected patients with MASLD in the general population. Nevertheless, limited data from studies of hepatic fibrosis do not support the notion that lower rates of hepatic steatosis translate into lower rates of hepatic fibrosis in acromegaly. Because progression from hepatic steatosis to fibrosis is a long–term pathogenic process, management of acromegaly and/or other factors may mask the natural history of the liver disease in acromegaly. Although this seemingly paradoxical finding could be clarified by cohort studies evaluating the natural history of MASLD in patients with acromegaly, such studies are difficult to conduct because follow–up of patients with untreated acromegaly raises ethical considerations.

## Management of Acromegaly and MASLD

Studies reporting on the long–term effects of acromegaly treatment (pharmacological and/or surgical) on MASLD are summarized in Table 2 [40, 41, 44, 45, 53-58]. All retrieved studies were case series, except for one open–label randomized controlled trial (RCT), largely owing to the rarity of acromegaly. Accordingly, small sample sizes are a notable limitation of all relevant studies. The study populations were drawn mainly from Europe and North America, with one study from Asia (Table 2). The first relevant study was published in 2005 [53]. Importantly, IHL was quantified using MRS in most studies, whereas abdominal ultrasound and a NIT (hepatic steatosis index [HSI]) were used in one study [56], and no imaging modality was used in another [53] (Table 2). Liver biopsy was not performed in any study.

**Table 2:** Effect of acromegaly treatment (pharmacological and surgical) on MASLD based on clinical studies*

First author Year Origin [Reference]†	Study design	Patients (*n*)	Medication; treatment duration^§^	Main findings^§^
Wiesli 2005 Switzerland [53]	Single center, case series; no imaging of the liver	Patients with acromegaly subjected to pituitary surgery (16)	Adenomectomy; follow–up: 4 weeks	ALT, glucose, insulin and HOMA–IR decreased, and adiponectin increased after adenomectomy. Body weight and waist circumference did not change after adenomectomy.
Madsen 2012 Denmark [54]	Single center, open–label RCT; IHL was evaluated with MRS	Patients with controlled acromegaly on SA monotherapy (18): combination group (12) vs. SA monotherapy (unchanged; 6)	Pegvisomant (15–30 mg twice a week) and SA (half dose) vs. SA monotherapy (unchanged); 24 weeks	IHL increased in the combination group (1.4 ± 0.5), whereas decreased (-3.6 ± 1.6) in the SA group (between groups *p* = 0.002). The increase in IHL was positively correlated with the weekly pegvisomant dose, but not with IR or circulating markers of inflammation.
Winhofer 2014 Austria [40]	Single center, case series; IHL was evaluated with MRS	Patients with controlled acromegaly (7)	Adenomectomy; follow–up: 6 months	IHL did not change after adenomectomy.
Reyes-Vidal 2015 USA [55]	Single center, case series; IHL was evaluated with MRS	Patients with untreated (active) acromegaly (23)	Adenomectomy; follow–up: 2 years	IHL increased after adenomectomy. VAT and SAT mass increased, whereas skeletal muscle mass and HOMA–IR decreased after adenomectomy. Body weight and waist circumference did not change after adenomectomy.
Bredella 2017 USA [41]	Single center, case series; IHL was evaluated with MRS	Patients with active acromegaly (16)	Adenomectomy and/or SA; follow–up: 8 (3–22) months^ƒ^	IHL increased after the control of acromegaly [from 2.0 (1.0–4.0) to 3.0 (1.0–3.0)%]^#^. VAT and SAT mass increased, whereas skeletal muscle mass, glucose, insulin and HOMA–IR decreased after the control of acromegaly. BMI did not change after the control of acromegaly. Similar body composition between patients on and not on SA.
Ciresi 2018 Italy [56]	Single center, case series; HS was evaluated with US and HSI	Patients with active acromegaly that refused adenomectomy (31)	SA; 12 months	Based on US, HS remained the same after treatment (from 61.2 to 61.2% of patients). Based on HSI, HS decreased after treatment (from 41.6 ± 5 to 35.2 ± 4.3). Insulin and HOMA–IR decreased after treatment. BMI, waist circumference, LFTs and lipid profile did not change after treatment.
Xie 2018 China [57]	Single center, case series; IHL was evaluated with MRS	Patients with acromegaly (17)	Adenomectomy; follow–up: 1 year	IHL increased, after a transient decrease at month 1, after adenomectomy [from 10.5 (baseline) to 9.4 (month 1), 10.9 (month 3), 12.1 (month 6), 18.2% (month 12)]. AST and ALT increased after adenomectomy. VAT and abdominal SAT mass increased after adenomectomy. BMI did not change after adenomectomy.
Kuker 2021 USA [44]	Single center, case series; IHL was evaluated with MRS	Patients with active acromegaly (8 of 21 subjected to hepatic MRS)	Pegvisomant monotherapy [20 (5–40 mg/day]^ƒ^; follow–up: 5.7 (1.0–13.4) years^ƒ^	IHL increased after pegvisomant treatment [from 1.8 (0.7–5) to 3.5 (1.6–10.6%)]^ƒ^. VAT and SAT mass increased after pegvisomant treatment. Waist circumference, but not BMI, increased after pegvisomant treatment. HOMA–IR decreased, whereas leptin increased after pegvisomant treatment.
Arlien-Søborg 2023 Denmark [45]	Single center, case series; IHL was evaluated with MRS	Patients with untreated (active) acromegaly (21)	Adenomectomy and/or SA and/or GHR antagonist; follow–up: 44 (16–163) weeks^∂^	IHL increased after the control of acromegaly [from 1.8 (1.0–3.2) to 3.0 (1.6–5.4)%]^∂^. IHL increased more after pharmacological treatment than adenomectomy; this was attributed mainly to GHR antagonists. Glucose, insulin, HOMA–IR and LBM decreased, whereas TBF increased after the control of acromegaly.
Kuker 2023 USA [58]	Single center, case series; IHL was evaluated with MRS	Patients with active acromegaly (28)	Adenomectomy; follow–up: 2.3 ± 1.3 years	IHL increased after adenomectomy (from 1.2 ± 1.4 to 4.0 ± 5.4%). Changes in IHL were not correlated with changes in IR.

*ALT* alanine aminotransferase, *AST* aspartate aminotransferase, *BMI* body mass index, *CI* confidence interval, *GHR* growth hormone receptor, *HOMA–IR* homeostasis model assessment–insulin resistance, *HS* hepatic steatosis, *HSI* hepatic steatosis index, *IHL* intrahepatic lipid, *IR* insulin resistance, *LBM* lean body mass, *LFTs* liver function tests, *MAFLD* metabolic dysfunction–associated fatty liver disease, *MASLD* metabolic dysfunction–associated steatotic liver disease, *MRS* magnetic resonance spectroscopy, *n* number, *NAFLD* nonalcoholic fatty liver disease, *SA* somatostatin analog, *SAT* subcutaneous adipose tissue, *SD* standard deviation, *TBF* total body fat, *US* ultrasonography, *VAT* visceral adipose tissue

*: Original nomenclature of the liver disease (i.e., NAFLD, MAFLD, MASLD) was retained in the table for each study, for the sake of consistency

†: Studies are sorted in alphabetical order, primarily according to the publication year and secondarily according to the first author surname

§: Data presented as mean ± standard deviation (SD), except if it is differently indicated

∂: Median (95% CI)

ƒ: Median (range)

#: Median (25th 75th percentile)

### Effects of Surgical Treatment of Acromegaly on MASLD

In all but one study [40], adenomectomy of a GH–secreting tumor led to higher IHL after approximately 1.0–2.5 years of follow–up [41, 45, 55, 57, 58]. Notably, BMI did not change [41, 53, 55, 57], but VAT and subcutaneous adipose tissue (SAT) increased [55]. Conversely, after pituitary adenomectomy, lean mass, including skeletal muscle mass, decreased [41, 45, 55, 57]. This redistribution between adipose and skeletal muscle mass may result in essentially unchanged BMI after pituitary adenomectomy. Therefore, BMI does not appear to be a good index of visceral adiposity after acromegaly remission. It is of interest that IHL and VAT increased after pituitary adenomectomy [41, 45, 55, 57], despite a simultaneous decrease in IR, as assessed with homeostasis model assessment–insulin resistance (HOMA–IR) [41, 45, 53]. In line with this observation, changes in IHL were not associated with changes in IR [58]. Therefore, although IR is a major driver of hepatic steatosis [16], GH excess may outweigh any IR-mediated effect on hepatic steatosis in patients with acromegaly. Additionally, when GH excess is successfully managed, IR decreases, but IHL increases, largely irrespective of IR.

### Effects of Pharmacological Treatment of Acromegaly on MASLD

Limited data are available on the effects of SA and/or pegvisomant, a GH receptor antagonist, on MASLD in patients with acromegaly (Table 2). In patients with controlled acromegaly, the combination of SA and pegvisomant increased IHL more than SA monotherapy [54]. In this study, the increase in IHL was positively associated with pegvisomant dose but not with IR [54], implying that GH receptor inhibition may be a stronger correlate of hepatic steatosis than IR. Pegvisomant monotherapy also increased IHL in patients with active acromegaly; as with pituitary adenomectomy mentioned above, pegvisomant treatment increased VAT and SAT (without affecting BMI) and decreased HOMA–IR [44]. Overall, these findings suggest that treatment effects on IHL may primarily be associated with the control of GH excess rather than with the type of treatment (adenomectomy or pharmacological). In another case series, pharmacological treatment, mainly pegvisomant, was reported to increase IHL more than adenomectomy in patients with untreated acromegaly; however, the comparative effect of pharmacotherapy vs. adenomectomy on hepatic steatosis warrants further investigation in studies of different design [45]. However, RCTs are hardly feasible in this case, because randomly allocating patients with acromegaly to adenomectomy or pharmacotherapy may raise ethical concerns. Moreover, SA monotherapy did not affect the rates of hepatic steatosis assessed by ultrasonography in another case series; however, HSI showed decreased hepatic steatosis after treatment, yielding conflicting results [56]. Notably, in a case–control study, HSI was not correlated with hepatic steatosis assessed by MRI–PDFF among patients with active or controlled acromegaly [46], suggesting that HSI application in patients with acromegaly may require careful interpretation. Notably, HOMA–IR also decreased, whereas BMI did not change after treatment [56]. Another observation is that circulating levels of adiponectin, an adipokine beneficial in MASLD [59], increased after pituitary adenomectomy [45, 53], but decreased after pharmacological treatment with SA or pegvisomant [45]. Other authors have previously reported that SA decreases circulating adiponectin concentrations in both lean and obese individuals without acromegaly [60, 61]. Although these studies cannot demonstrate distinct effects of SA vs. pegvisomant or pituitary adenomectomy, these observations warrant head–to–head comparative studies using a better imaging modality (e.g., MRI–PDFF or MRS) or, ideally, liver histological confirmation to definitively establish them.

It is also worth noting that a crossover study in 10 healthy volunteers who received pegvisomant and recombinant human GH (rhGH), with a washout period of at least 6 weeks, showed that pegvisomant doubled hepatic de novo lipogenesis; however, this effect was not statistically significant, likely due to the small sample size. Conversely, rhGH stimulated VLDL secretion from the liver, thereby reducing hepatic lipid burden [62].

### Safety of Pharmacological Treatment of Acromegaly: Focus on Liver-Related Issues

Regarding safety, a hepatic–related adverse effect of pegvisomant is an increase in LFTs, observed in approximately 5–8% of patients after treatment [63]. This LFT increase appears higher when pegvisomant is combined with SA and is reversible after pegvisomant withdrawal [63]. The pathophysiology of the pegvisomant–induced increase in LFTs remains largely unknown. However, this increase may be associated with the above–mentioned increase in IHL after pegvisomant treatment, but drug–induced liver injury (DILI) should also be considered. Clinical practice guidelines from the Endocrine Society recommend monitoring LFTs monthly for the first 6 months, then every 6 months, in patients with acromegaly receiving pegvisomant. Discontinuation of pegvisomant should be considered if LFTs exceed 3 times the upper limit [64].

Notably, SA exerts a dual effect on glucose homeostasis. On the one hand, SA decreases IR; on the other hand, it impairs pancreatic insulin secretion, resulting in glucose intolerance [65]. Consequently, circulating glucose levels may increase after SA treatment in patients with acromegaly, irrespective of disease control. By contrast, this is not observed in patients with disease remission after pituitary adenomectomy [66] or after pegvisomant treatment [13]. Of note, the rate of new–onset T2DM among patients with acromegaly receiving long–term (i.e., 5–year) SA treatment was approximately 15% [67]. This dual effect of SA may suggest that the drug, at least in part, increases hepatic steatosis by adversely affecting pancreatic insulin secretion, which predisposes some individuals to develop T2DM despite decreasing IR. This intriguing effect of SA treatment on hepatic steatosis warrants further investigation. It should also be noted that long-acting pasireotide, a somatostatin multi-receptor ligand, is effective in achieving biochemical control in acromegaly but adversely affects glucose metabolism, leading to high rates of T2DM after treatment [68]. Although the effect of pasireotide on MASLD has not yet been specifically evaluated, its adverse effect on glucose metabolism may be associated with higher rates of MASLD.

### Critical Summary of the Association of Acromegaly Management with MASLD

Existing data suggest that treatment of acromegaly, whether surgical or pharmacological, increases hepatic steatosis, whereas few data exist on the effects of treatment on hepatic inflammation and fibrosis. It seems that control of GH excess can lead to redistribution of body composition, characterized by decreased skeletal muscle mass and increased VAT and SAT (i.e., changes that resemble sarcopenic obesity [69]), as well as IHL, which occur despite the concomitant decrease in IR (Table 2). Theoretically, management of acromegaly, by increasing hepatic steatosis, may lead to increased hepatic inflammation and fibrosis in the long term, and by decreasing IGF–1, may diminish the suppression of HSCs, thereby increasing hepatic fibrosis. However, these effects on hepatic inflammation and fibrosis have not yet been demonstrated. 

Secondary effects of pegvisomant on LFTs and SA on glucose metabolism, which may also affect hepatic steatosis, should be considered and warrant further investigation. Of course, these effects must not discourage acromegaly treatment, because acromegaly is a chronic, debilitating disease that leads to poor quality of life and reduced survival, as a result of multisystem morbidity [13]. However, relevant research should focus on strategies to minimize these adverse effects of disease control on MASLD, as well as on sarcopenic obesity. It may be hypothesized that complete pituitary adenomectomy by expert neurosurgeons, resulting in the cure of acromegaly and thereby eliminating the need for additional pharmacological treatment, followed by intensive lifestyle interventions, may be a rational therapeutic approach.

## Closing Remarks

This narrative review summarizes the evidence regarding the association between acromegaly and MASLD in adults. Active (uncontrolled) acromegaly is characterized by IR despite low VAT and IHL (Table 1), reflecting an intriguing dissociation of IR from visceral obesity and hepatic steatosis. It has also been suggested that intramuscular lipid deposition (myosteatosis) is increased in skeletal muscle [15], but not in the myocardium [40]. Successful surgical or pharmacological control of acromegaly reverses this metabolic profile, leading to reduced IR but increased VAT and hepatic steatosis (Table 2). This suggests that the dissociation of IR from visceral obesity and hepatic steatosis persists even after disease management. The reason for this paradoxical dissociation remains largely unknown. GH excess appears to affect both IR and MASLD, but in opposite directions. In this regard, any indirect IR–mediated effect on hepatic steatosis appears to be smaller than the direct GH excess–mediated effect on hepatic steatosis in patients with acromegaly. Some authors have suggested that this may be partly due to increased hepatic mitochondrial activity, which leads to enhanced hepatic fatty acid β–oxidation and energy expenditure observed in patients with active acromegaly, thereby counteracting hepatic lipid accumulation. Consequently, body composition changes, with lean body mass and body water increasing, and VAT and SAT decreasing [40, 43]. Another plausible explanation is that enhanced intramuscular lipid deposition may contribute to the high IR observed in acromegaly [55, 70].

An area of concern is the effect of acromegaly management on hepatic inflammation and fibrosis, as well as on HCC, all of which are poorly studied. On the one hand, acromegaly management can lead to hepatic steatosis, so, based on the natural history of MASLD, higher rates of hepatic inflammation and fibrosis might be expected. On the other hand, acromegaly management also lowers IR, a major driver of MASLD progression, which may counterbalance the effect of hepatic steatosis in advanced MASLD. Therefore, long–term prospective studies in patients with acromegaly after disease control are warranted to elucidate this intriguing topic. Despite experimental data favoring long–term oncogenic effects of GH excess in the liver, the clinical association between acromegaly management and MASLD–related HCC is difficult to investigate, partly owing to the rarity of acromegaly and the low prevalence of HCC in long–standing MASLD. 

Patients with MASLD [6, 7] or acromegaly [13] have multimorbidity, meaning both diseases affect multiple organs and are therefore considered multisystem diseases. In both MASLD [71] and acromegaly [72], the leading causes of mortality are CVD and malignancies. Specifically, CVD is the leading cause of mortality in MASLD, followed by extrahepatic malignancies [71], whereas in acromegaly, there has been a shift over the last two decades, with hepatic and extrahepatic malignancies becoming the leading cause of mortality, followed by CVD [72]. Based on the data in this review, we could not support the notion that MASLD increases CVD or cancer–related mortality in individuals with acromegaly, as observed in the general population or in specific populations with MASLD [73], because the rates of hepatic steatosis are low and the rates of hepatic fibrosis are largely unstudied in active acromegaly. However, because the rates of hepatic steatosis increase after treatment of acromegaly, longitudinal studies, ideally prospective ones, are required to evaluate the long–term effect of controlling acromegaly on CVD and cancer–related morbidity and mortality. Until then, it is conceivable that the benefits of controlling acromegaly may outweigh any adverse effects on MASLD with respect to CVD and cancer–related morbidity and mortality in patients with acromegaly.

Current data do not support the development of guidelines for MASLD in patients with acromegaly or the implementation of MASLD screening in this population. Personalized management of patients with acromegaly is required to provide the most appropriate treatment, surgical and/or pharmacological, with the latter at the lowest doses that control the disease, to avoid adverse effects on MASLD (Table 2), as well as other relevant adverse effects, including DILI after pegvisomant treatment [63] or glucose intolerance/T2DM [65] after SA treatment. Furthermore, strict follow–up with appropriate counseling is likely needed to guide patients with controlled acromegaly in lifestyle changes (diet and physical activity) [74], aiming to minimize the effects of acromegaly control on VAT and hepatic steatosis. Special attention may be needed to avoid post–surgical GHD, e.g., in cases of extensive adenomectomy, which may also lead to adverse effects in terms of MASLD, visceral obesity, and CVD morbidity and mortality [10]. It is also noted that the intriguing associations of high IR with low visceral adiposity and low rates of hepatic steatosis observed in patients with acromegaly cannot be extrapolated to the general adult population, in whom high IR is usually associated with high visceral adiposity and high rates of hepatic steatosis [16].

In conclusion, patients with acromegaly have lower rates of hepatic steatosis despite high IR. Surgical or pharmacological treatment of acromegaly increases hepatic steatosis and decreases IR, although the long–term effects of treatment on hepatic inflammation, fibrosis, and HCC remain largely unknown. Given the intriguing associations among acromegaly, MASLD and IR, further clinical and mechanistic studies are needed to clarify their pathophysiological links and their clinical and therapeutic implications.

## Data Availability

No datasets were generated or analysed during the current study.

## Declarations

### AI statement

No AI tool was used in this article.

### Originality of figures and tables

All figures and tables are original and were developed specifically for this article; therefore, no permission to reproduce is required.
